# Supplementary material for: The diagnostic test accuracy of telemedicine for detection of surgical site infection: A systematic review protocol
Source: PLoS One. 2022 Nov 17;17(11):e0263549. doi: 10.1371/journal.pone.0263549 (PMC9671442; doi:10.1371/journal.pone.0263549)
Supplement: S2 Table — The ASEPSIS scoring method. A score of ≥21 equates to the presence of SSI. (DOCX) [file pone.0263549.s002.docx]

## S2 Table: Asepsis Score

| **Scores awarded for ASEPSIS components** | |
| --- | --- |
|  | **Score** |
| **Wound characteristic** | |
| Serous exudates | 3 |
| Erythema | 3 |
| Purulent exudates | 6 |
| Separation of wound edges | 6 |
| **Additional treatment** | |
| Postoperative Antibiotics | 10 |
| Abscess drainage | 5 |
| Wound debridement | 10 |
| Isolation of bacteria | 10 |
| Prolonged stay/admission to hospital | 5 |

**An ASEPSIS score of ≥21 is taken as indicating the presence of infection, whilst a score of ≤ 10 is taken to represent satisfactory healing.**
