# Supplementary material for: The diagnostic test accuracy of telemedicine for detection of surgical site infection: A systematic review protocol
Source: PLoS One. 2022 Nov 17;17(11):e0263549. doi: 10.1371/journal.pone.0263549 (PMC9671442; doi:10.1371/journal.pone.0263549)
Supplement: S1 File — SSI classification into superficial incisional, deep incisional, organ/space infection. (DOCX) [file pone.0263549.s003.docx]

## S1 File: SSI Classification

*Superficial incisional infection*

Defined as a surgical site infection occurring within 30 days of surgery and involves only the skin or subcutaneous tissue of the incision, and meets at least one of the following criteria:

Criterion 1: Purulent drainage from the superficial incision.

Criterion 2: The superficial incision yields organisms from the culture of aseptically aspirated fluid or tissue, or from a swab and pus cells are present.

Criterion 3: At least two of the following symptoms and signs:

- pain or tenderness

- localised swelling

- redness

- heat

AND a. the superficial incision is deliberately opened by a surgeon to manage the infection, unless the incision is culture-negative

OR b. the clinician diagnoses a superficial incisional infection.

Note: Stitch abscesses are defined as minimal inflammation and discharge confined to the points of suture penetration, and localised infection around a stab wound. They are not classified as surgical site infections.

*Deep incisional infection*

Defined as a surgical site infection involving the deep tissues (i.e. fascial and muscle layers) that occurs within 30 days of surgery if no implant is in place, or within 90 days if an implant is in place and the infection appears to be related to the surgical procedure, and meets at least one of the following criteria:

Criterion 1: Purulent drainage from the deep incision but not from the organ/space component of the surgical site.

Criterion 2: The deep incision yields organisms from the culture of aseptically aspirated fluid or tissue, or from a swab and pus cells are present.

Criterion 3: A deep incision that spontaneously dehisces or is deliberately opened by a surgeon when the patient has at least one of the following symptoms or signs (unless the incision is culture negative):

- fever (>38^o^C)

- localised pain or tenderness

Criterion 4: An abscess or other evidence of infection involving the deep incision that is found by direct examination during re-operation, or by histo-pathological or radiological examination.

Criterion 5: Diagnosis of a deep incisional surgical site infection by an attending clinician.

Note: An infection involving both superficial and deep incision is classified as deep incisional SSI unless there are different organisms present at each site.

*Organ/space infection*

Defined as a surgical site infection involving any part of the anatomy (i.e. organ/space), other than the incision, opened or manipulated during the surgical procedure, that occurs within 30 days of surgery if no implant is in place, or within 90 days if an implant is in place and the infection appears to be related to the surgical procedure, and meets at least one of the following criteria:

Criterion 1: Purulent drainage from a drain that is placed through a stab wound into the organ/space.

Criterion 2: The organ/space yields organisms from the culture of aseptically aspirated fluid or tissue, or from a swab and pus cells are present.

Criterion 3: An abscess or other evidence of infection involving the organ/space that is found by direct examination, during re-operation, or by histo-pathological or radiological examination.

Criterion 4: Diagnosis of an organ/space infection by an attending clinician

Note: 1. Occasionally, an organ/space infection drains through the incision. Such infection generally does not require re-operation and is considered to be a complication of the incision, and is therefore classified as a deep incisional infection.

2. Where doubt exists, refer to the Definitions of specific site of organ/space infection to determine if the organ/space infection meets the definition
