# Supplementary material for: The diagnostic test accuracy of telemedicine for detection of surgical site infection: A systematic review protocol
Source: PLoS One. 2022 Nov 17;17(11):e0263549. doi: 10.1371/journal.pone.0263549 (PMC9671442; doi:10.1371/journal.pone.0263549)
Supplement: S2 File — The QUADAS-2 tool for assessment of methodological quality of studies. (DOCX) [file pone.0263549.s004.docx]

## S2 File: QUADAS-2

The process occurs in four phases;

1. Statement of the review question

This involves a summary of the review at hand in the form of patients, index tests, reference standards and target condition, details of which can be found in sections 2.2.2 – 2.2.5.

1. Validation of QUADAS-2 tool

Both authors will utilise the tool in two example papers to be included as suggested in the QUADAS-2 supporting material. Agreement of 80% or more across all categories will be taken as sufficient and QUADAS-2 taken forward for assessment of all studies. Insufficient agreement will lead to further refinement of the tool through addition or omitting signalling questions and the process repeated until agreement is satisfactory.

1. Flow diagram

The primary study’s flow diagram will be taken (or synthesised if not provided) to facilitate judgments of risk of bias in phase four. This will provide information on the method of patient recruitment, the order of test execution, the number of patients undergoing the index test and reference standard

1. Judgments on bias and applicability

*Risk of Bias*

The tool itself comprises four separate domains, the first section of each concerns bias and has three sections.

- Information used to support the risk of bias judgment; to make the rating transparent and can aid discussion between independent review authors
- Signalling questions; presented to assist judgements. Answered ‘yes’, ‘no’, or ‘unclear’ so that ‘yes’ would indicate a low risk of bias.
- Judgement of the risk of bias; ‘low’, ‘high’, or ‘unclear’. If all signalling questions are answered ‘yes’ then risk of bias is to be judged as ‘low’. If any signalling question returns ‘no’ there would be potential for bias. The guidelines from phase two will assist authors in the judgement. ‘Unclear’ should only be used if insufficient data are reported to allow a judgement.

*Applicability*

These sections do not include signalling questions. Authors should record information on which the applicability judgement is made and then rate their concern that the study does not match the review question. This can be rated as ‘low’, ‘high’ or ‘unclear’. Again, ‘unclear’ is only to be used when insufficient data are available.

Guidance for completing the QUADAS-2 tool

**Domain 1: Patient Selection**

*Risk of bias: Could the selection of patients have introduced bias?*

- Was a consecutive or random sample of patients enrolled?
- Was a case control design avoided?
- Did the study avoid inappropriate exclusions?

Ideally studies should enrol all consecutive or a random sample of patients undergoing surgery, otherwise there is potential for bias. Inappropriate exclusions (difficult diagnosis) may skew estimates of diagnostic accuracy. Enrolment of patients with known SSI may also exaggerate diagnostic accuracy.

*Applicability: Are there concerns that the included patients and setting do not match the review question?*

Patients included in the study should match those in the review question to ensure applicability of results. This can be in terms of SSI severity, demographics, comorbidity, study setting.

**Domain 2: Index Text**

*Risk of Bias: Could the conduct or interpretation of the index test have introduced bias?*

- Were the index test results interpreted without knowledge of the results of the reference standard?
- If a threshold was used, was it pre-specified

The first signalling question refers to blinding, and the potential for bias with regards to testing order. If the index test is always conducted and interpreted prior to the reference standard, then this item can be rated ‘yes’.

The second question depends on the method of telemedicine used. If bluebelle WHQ is used in any of the methods this should be indicated what the cut off for SSI is.

*Applicability: Are there concerns that the index test, it’s conduct, or interpretation differ from the review question?*

Variations in test technology, execution or interpretation may affect estimates of it’s diagnostic accuracy. If these vary from those specified in the review question there may be concerns of applicability.

**Domain 3: Reference Standard**

*Risk of Bias: Could the reference standard, its conduct, or its interpretation have introduced bias?*

- Is the reference standard likely to correctly classify the target condition?
- Were the reference standard results interpreted without knowledge of the results of the index test?

What version of the reference standard was used? CDC / ASEPSIS / Bluebelle / other? How likely is that to have correctly identify SSI?

Potential for bias is related to the potential influence of prior knowledge on the interpretation of the reference standard, similar to that in domain two.

*Applicability: Are there concerns that the target condition as defined by the reference standard does not match the question?*

The target condition, SSI, defined by the reference standard, may differ from the SSI specified in the review question.

**Domain 4: Flow and Timing**

*Risk of Bias: Could the patient flow have introduced bias?*

- Was there an appropriate interval between index test and reference standard?

Ideally results on the index test and reference standard are collected on the same patient at the same time. Delay or initiation of treatment between one of the two tests may result in misclassification due to recovery or deterioration in SSI criteria.

- Did all patients receive the same reference standard?

Verification bias occurs when not all the study patients receive confirmation of diagnosis by the same reference standard.

- Were all patients included in the analysis?

All patients recruited into the study should be included in the analysis. If the number of patients enrolled differs from the number of patients included in the 2x2 table of results, there is potential for bias.

Presentation of QUADAS-2 results

Overall generalisable statements of ‘low’ or ‘high risk of bias’ will not be used unless a study is judged to be ‘low’ across all domains or if the study has been judged ‘high’ or ‘unclear’ in one or more domains it may be at risk of bias or as having concerns regarding applicability.

Results will be presented in the following cross tabulation whereby ‘+’ indicates high risk or concern, ‘-‘ indicates low risk or concern and ‘?’ indicates unclear.

| Study | Risk of bias | | | | Applicability Concerns | | |
| --- | --- | --- | --- | --- | --- | --- | --- |
|  | Patient Selection | Index Test | Reference Standard | Flow and Timing | Patient Selection | Index Test | Reference Standard |
| Study 1 | + / - / ? | + / - / ? | + / - / ? | + / - / ? | + / - / ? | + / - / ? | + / - / ? |
| Study 2 | + / - / ? | + / - / ? | + / - / ? | + / - / ? | + / - / ? | + / - / ? | + / - / ? |
| Study 3 | + / - / ? | + / - / ? | + / - / ? | + / - / ? | + / - / ? | + / - / ? | + / - / ? |
| … | + / - / ? | + / - / ? | + / - / ? | + / - / ? | + / - / ? | + / - / ? | + / - / ? |

Table 1: Representation of tabulation for QUADAS-2 analysis on systematic review studies.

Recommended quality items derived from QUADAS tool

| 1. | Was the spectrum of patients representative of the patients who will receive the test in practice? (representative spectrum) |
| --- | --- |
| 2. | Is the reference standard likely to classify the target condition correctly? (acceptable reference standard) |
| 3. | Is the time period between reference standard and index test short enough to be reasonably sure that the target condition did not change between the two tests? (acceptable delay between tests) |
| 4. | Did the whole sample or a random selection of the sample, receive verification using the intended reference standard? (partial verification avoided) |
| 5. | Did patients receive the same reference standard irrespective of the index result? (differential verification avoided) |
| 6. | Was the reference standard independent of the index test (i.e the index test did not form part of the reference standard) incorporation avoided) |
| 7. | Were the reference standard results interpreted without knowledge of the results of the index test? (index test results blinded) |
| 8. | Were the index test results interpreted without knowledge of the results of the reference standard (reference standard results blinded) |
| 9. | Were the same clinical data available when test results were interpreted as would be available when the test is used in practice? (relevant clinical information) |
| 10. | Were uninterpretable/intermediate test results reported? (uninterpretable results reported) |
| 11. | Were withdrawals from the study explained? (withdrawals explained) |
|  | Additional items |
| 12. | Is the technology of the index test unchanged since the study was carried out? |
| 13. | Did the study provide a clear definition of what was considered to be a positive result? |
| 14. | Were data on observer variation reported and within an acceptable range? |
| 15. | Was treatment withheld until both the index test and reference standard were performed? |
| 16. | Were objectives pre-specified? |
